# Objective risk assessment using a driving computer game

**DOI:** 10.1192/j.eurpsy.2021.1361

**Published:** 2021-08-13

**Authors:** D. Delgado-Gomez, A. Sujar, J. Ardoy-Cuadros, S. Moana, I. Peñuelas-Calvo, D. Aguado, H. Blasco-Fontecilla

**Affiliations:** 1 Department Of Statistics, Universidad Carlos III, Getafe, Spain; 2 Department Of Psychiatry, Puerta de Hierro University Hospital, Majadahonda, Spain; 3 Department Of Psychology, University Rey Juan Carlos, Alcorcon, Spain; 4 Department Of Child And Adolescent Psychiatry, Hospital Universitario Fundación Jiménez Díaz, Madrid, Spain; 5 Department Of Social Psychology And Methodology, Autonoma University of Madrid, Madrid, Spain

**Keywords:** Self-report of Risk-taking Behaviors, risk, video games, e-health

## Abstract

**Introduction:**

Accurate and objective risk assessment is important in the evaluation of many mental disorders and behaviours. For example, in the evaluation of suicidal behaviour or the assessment of accidents in ADHD. Video games could contribute to improve the assessment and increase engagement.

**Objectives:**

Our hypothesis is that the proposed videogame can precisely evaluate risk. In addition, the developed game is able to indirectly assess the risk. This feature is useful in setups where patients are prone to lie.

**Methods:**

We have developed a car driving video game where users are told that they should drive near to the border but not too much. We record distance to the border and each key pulsation every 0.1 seconds.

**Figure:**
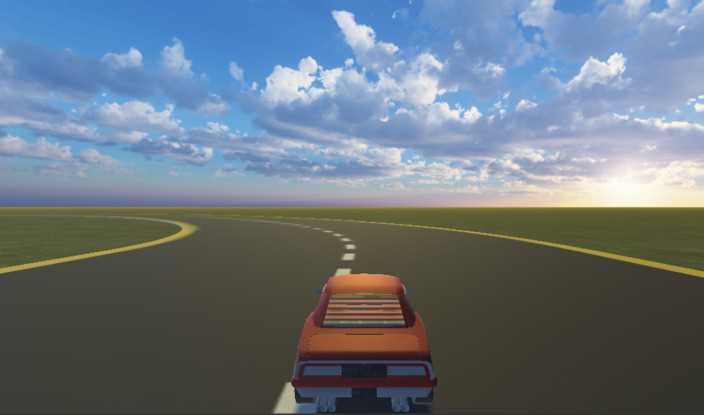

**Results:**

It has been observed that the median of recorded distance positively correlated with the score obtained by Self-report of Risk-taking Behaviors (SRB). In addition, the interquartile range significant correlates with the global score obtained in this questionnaire.

**Conclusions:**

The proposed videogame is able of performing an accurate risk assessment. Our game takes seven minutes and it does not need complicated nor expensive hardware and could be deployed online. Results obtained open up new possibilities of creating video games which make an objective assessment risk.

